# Expanding the Editing Window of Cytidine Base Editors With the Rad51 DNA-Binding Domain in Rice

**DOI:** 10.3389/fpls.2022.865848

**Published:** 2022-04-25

**Authors:** Chunjie Wei, Hao Liu, Wenwen Wang, Pengyu Luo, Qiuling Chen, Rou Li, Chong Wang, José Ramón Botella, Hui Zhang

**Affiliations:** ^1^Shanghai Key Laboratory of Plant Molecular Sciences, Development Center of Plant Germplasm Resources, College of Life Sciences, Shanghai Normal University, Shanghai, China; ^2^School of Agriculture and Food Sciences, University of Queensland, Brisbane, QLD, Australia

**Keywords:** ssDBD, Rad51 DBD, CBEs, editing window, rice

## Abstract

Recently developed base editors provide a powerful tool for plant research and crop improvement. Although a number of different deaminases and Cas proteins have been used to improve base editors the editing efficiency, and editing window are still not optimal. Fusion of a non-sequence-specific single-stranded DNA-binding domain (DBD) from the human Rad51 protein between Cas9 nickase and the deaminase has been reported to dramatically increase the editing efficiency and expand the editing window of base editors in the mammalian cell lines and mouse embryos. We report the use of this strategy in rice, by fusing a rice codon-optimized human Rad51 DBD to the cytidine base editors AncBE4max, AncBE4max-NG, and evoFERNY. Our results show that the addition of Rad51 DBD did not increase editing efficiency in the major editing window but the editing range was expanded in all the three systems. Replacing the human Rad51 DBD with the rice Rad51 DBD homolog also expanded the editing window effectively.

## Introduction

Many important agronomic traits are controlled by single nucleotide polymorphisms (SNPs) and incorporation of beneficial SNPs into elite lines by traditional breeding is a long and tedious process. In recent years, CRISPR-based genome editing technologies have provided revolutionary tools allowing to incorporate SNPs in a fast and efficient way. The “basic” CRISPR/Cas9 system produces double-stranded breaks (DSBs) with high-target precision, which mainly leads to either small base insertions, deletions (indels), or substitutions although the nature of the changes are random (Hilton and Gersbach, 2015; Zhang et al., 2017). The development of base editors (BEs) by fusing a deaminase to a Cas9 nickase, has provided a more reliable and efficient method to generate SNPs. According to the nature of the deaminase, BEs are classified into adenosine base editors (ABEs) and cytidine base editors (CBEs). The ABEs can generate site-specific A⋅T to G⋅C substitutions while the CBEs can produce site-specific C⋅G to T⋅A substitutions (Komor et al., 2016; Gaudelli et al., 2017; Rees and Liu, 2018).

From the initial, relatively inefficient BEs, several developments have increased the editing efficiency, expanded the targeting scope, and improved the editing specificity (Anzalone et al., 2020). CBEs have undergone improvements through several generations, from BE1 to BE4max, with average editing efficiencies increasing from 0.8 ∼ 7.7 to 65% (Komor et al., 2016, 2017; Koblan et al., 2018). Similarly, ABEs have evolved to increase editing efficiency (Gaudelli et al., 2017; Koblan et al., 2018; Richter et al., 2020). Most improvement approaches have focused on the improvement of deaminases (such as evoFERNY and TadA8e) and optimization of system components, such as the domain-linking sequences and nuclear localization signals (NLSs), achieving important gains in editing efficiency (Koblan et al., 2018; Thuronyi et al., 2019; Wei et al., 2021). However, the editing window of the base editors is still relatively narrow, limiting their usefulness in functional genomics research and crop improvement. The nature of the deaminase is the main contributor to the size of the editing window. For most cytidine deaminases, such as rAPOBEC1, hAID, hAPOBEC3A, and PmCDA1, the editing window spans the protospacer positions 4 (C4) to 8 (C8) (counting the PAM as positions 21–23). The A3A base editor, using the human APOBEC3A cytidine deaminase, exhibited an expanded editing window, from C1 to C17, in plants (Zong et al., 2018). The evolved deaminases evorAPOBEC1, evoFERNY, and evoCDA1 also expanded the editing window from positions C1 to C15 (Zeng et al., 2020b). Although some of the newly developed CBEs have a relatively large editing window, the editing efficiency for PAM-proximal sequences (protospacer positions 12–20) is still very low (Anzalone et al., 2020).

Fusion of a single-stranded DNA binding domain (DBD) from the Rad51 protein with CBEs has also been proven to increase the editing efficiency and broaden the editing window in animal and plant systems (Zhang et al., 2020; Xu et al., 2022). The Rad51 protein plays significant roles in eukaryotic DNA recombination, including homology search and DNA-strand exchange (Conway et al., 2004; Heyer et al., 2010). Fusion of the human Rad51 with the Cas9 (D10A) nickase successfully mediated recombination by the homology-directed repair (HDR) pathway (Rees et al., 2019). The Rad51 N-terminal domain contains 114 residues involved in DNA binding (Aihara et al., 1999) and several groups have successfully used this domain with CBEs and prime editors (PEs) to improve editing efficiency and expand the editing window (Zhang et al., 2020; Song et al., 2021). Fusion of the Rad51 DBD with the Nmcas9-based CBE, termed hyper CBE (hyCBE), has proven successful in rice. While no base editing was achieved using the Nm1-BE3 and Nm2-BE3 systems, the C-to-T conversion frequency for Nm1-hyBE3 was 12.5 and 10.4% at the NAL1-5 and PDS-6 targets respectively and 14.6% at the GRF6-1 target for Nm2-hyBE3 (Xu et al., 2022).

In this work, we fused the human Rad51 DBD to several CBEs including AncBE4max, AncBE4max-NG, and evoFERNY, and also fused the rice Rad51 DBD to AncBE4max and evoFERNY in an attempt to produce improved CBE systems for rice. Although the editing efficiency did not increase as expected, the editing window was expanded to C16. These new systems will be useful for the generation of SNPs, especially in saturation mutagenesis.

## Materials and Methods

### Materials and Growth Conditions

The *Oryza sativa* L. japonica varieties Zhonghua11 and Xiushui134 were grown in the paddy field in Shanghai, China, during the normal rice-growing season, and seeds were collected for callus production. All transgenic rice was grown in a rice greenhouse (12-h light 28°C and 12-h darkness at 22°C).

### Vector Constructions

Rice codon-optimized human Rad51 DBD (342-bp) and 96-bp linker (Wei et al., 2021) containing *BamH* I restriction sites at both ends were synthesized (TSINGKE, Shanghai, China) and cloned into the *BamH* I site of AncBE4max (Wang et al., 2019) between Anc689APOBEC and nCas9, to produce hyAncBE4max. For the construction of hyAncBE4max-NG, hyAncBE4max was digested with *BamH* I, and the human Rad51 DBD-linker fragment cloned into *BamH* I-digested AncBE4max-NG vector between Anc689APOBEC and nCas9-NG (Wang et al., 2019). The rice Rad51 DBD (342-bp) with a 96-bp linker containing *BamH* I restriction sites at both ends were synthesized (TSINGKE, Shanghai, China) and cloned into the *BamH* I site of AncBE4max between Anc689APOBEC and nCas9, to produce hyriceAncBE4max.

For the construction of the evoFERNY vector, the rice codon-optimized evoFERNY and the bipartite NLS (bpNLS) sequence were synthesized (TSINGKE, Shanghai, China) and cloned into the *Kpn* I/*BamH* I sites of the PUC57 vector, to produce PUC57-evoFERNY. PUC57-evoFERNY was digested with *Kpn* I/*BamH* I and the resultant bpNLS-evoFERNY fragment was cloned into the *Kpn* I/*BamH* I-predigested AncBE4max vector between the maize *Ubiquitin* (*ZmUbi*) promoter and nCas9(D10A). To produce the hyevoFERNY and hyriceevoFERNY vectors, the human Rad51 DBD-linker or rice Rad51 DBD-linker sequences were amplified using the specific primers (Supplementary Table 5) and cloned into the *BamH* I site of the binary vector evoFERNY using the ClonExpress MultiS One Step Cloning Kit (Vazyme, Nanjing, China).

For the construction of the target-specific base editing vectors, oligos containing the target-specific sequences and the adaptor sequences were synthesized and ligated to *Bsa* I-digested base editors, including AncBE4max, hyAncBE4max hyriceAncBE4max, AncBE4max-NG, hyAncBE4max-NG, evoFERNY, hyevoFERNY, and hyriceevoFERNY respectively. The final vectors were verified by Sanger sequencing using M13F as a primer. Primers used for vector construction are listed in Supplementary Table 5.

### Rice Transformation

Rice calli derived from seeds of Zhonghua11 and Xiushui134 were transformed by *Agrobacterium tumefaciens*-mediated transformation (Nishimura et al., 2006). Briefly, binary vectors were transferred into *Agrobacterium tumefaciens* EHA105 and were used to inoculate rice calli. After 48–60 h of co-cultivation with *Agrobacterium* at 25°C, the calli were moved to selection media containing 50 mg L^–1^ hygromycin for 1 month. Resistant calli were moved to medium containing 2 mg L^–1^ Kinetin (KT), 0.2 mg L^–1^ naphthalene acetic acid (NAA), and 50 mg L^–1^ hygromycin for regeneration. Regenerated shoots were moved to a medium containing 50 mg L^–1^ hygromycin for rooting.

### Genotyping of Transgenic Rice Lines

Genomic DNA was isolated from T0 generation rice leaves, derived from different tillers, using the cetyltrimethylammonium bromide (CTAB) method (Porebski et al., 1997). The M13F and sg-target-R primers were first used to identify positive transgenic lines and then the genomic regions surrounding the target sites were amplified using specific primers (Supplementary Table 5). PCR products were subjected to Sanger sequencing and subsequently analyzed using Geneious software to identify genotyping.

## Results

### Fusion of the Human Rad51 DNA-Binding Domain Expands the Editing Window of AncBE4max in Rice

**FIGURE 1:**
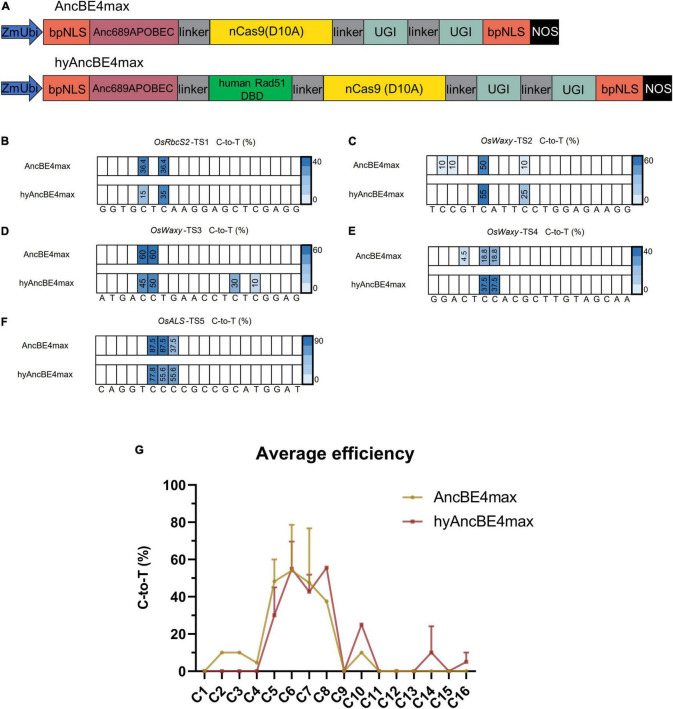
Characterization of AncBE4max and hyAncBE4max in rice. **(A)** Schematic structures of AncBE4max and hyAncBE4max. The human Rad51 DBD was synthesized using a plant optimized code and cloned into AncBE4max (Wang et al., 2019) to form hyAncBE4max. ZmUbi, maize *ubiquitin-1* promoter; bpNLS, bipartite nuclear localization signal; nCas9(D10A), nickase Cas9 with D10A substitution; UGI, uracil glycosylase inhibitor; NOS, NOS terminator. **(B–F)** C-to-T conversions in five target sites using AncBE4max or hyAncBE4max. **(G)** Average C-to-T editing efficiency for AncBE4max and hyAncBE4max based on all five target sites. Data are means ± SD.

The human Rad51 DBD was codon-optimized for rice using the online GenScript Codon Optimization Tool^1^ and fused to the AncBE4max to generate hyAncBE4max (Figure 1A). The AncBE4max chosen for our work contained the key elements of the Anc689 APOBEC deaminase, nickase Cas9 (nCas9) which recognizes NGG protospacer adjacent motif (PAM) sequences, two copies of the uracil glycosylase inhibitor (UGI), and two bipartite nuclear localization signals (bpNLS) at the N- and C-termini respectively (Figure 1A; Wang et al., 2019). The human Rad51 DBD was fused between the Anc689 APOBEC and nCas9 (D10A) with 32-amino acid linkers on each side (Figure 1A). Expression of the Anc689APOBEC/human Rad51 DBD/nCas9 cassette was driven by the maize *Ubiquitin* (*ZmUbi*) promoter while the rice *U6* promoter was used to drive the expression of the sgRNA (Figure 1A). Five target sites in three different genes were selected to analyze editing efficiency and editing window using the AncBE4max and hyAncBE4max systems (Supplementary Table 1). The binary vectors were used to transform rice callus *via Agrobacterium*-mediated infection and independent T0 transgenic lines genotyped by amplification of the target sequences followed by Sanger sequencing.

At the *OsRbcS2*-TS1 site, both AncBE4max and hyAncBE4max exhibited editing activity at positions C5 and C7 (Figure 1B and Supplementary Table 2). Although the C-to-T efficiency at position C7 was quite similar for both systems (AncBE4max 36.4 vs. hyAncBE4max 35%); contrary to our expectations, the efficiency of AncBE4max (36.4%) at position C5 was more than twice higher than that of hyAncBE4max (15%) (Figure 1B and Supplementary Table 2). At the *OsWaxy*-TS2 site, AncBE4max showed 10% editing efficiency at positions C2 and C3, while we failed to detect any editing activity for hyAncBE4max. At position C6 of the same target, both systems showed similar high C-to-T efficiency (50–55%), while at position C10, the C-to-T substitution efficiency was 10% for AncBE4max and 25% for hyAncBE4max (Figure 1C and Supplementary Table 2). At the *OsWaxy*-TS3 site, the substitution efficiency for hyAncBE4max and AncBE4max at the C5 and C6 positions were comparable (45 vs. 60% at C5, and 50 vs. 60% at C6) (Figure 1D and Supplementary Table 2). Nevertheless, the hyAncBE4max editing window was expanded to positions C14 and C16, with substitution efficiencies of 30.0% at position C14 and 10.0% at position 16 (Figure 1D). For the *OsWaxy*-TS4 site, AncBE4max produced 4.5% editing while hyAncBE4max showed no editing at position C4, while at positions C6 and C7, the C-to-T substitution efficiency was 18.8% for AncBE4max and 37.5% for hyAncBE4max (Figure 1E and Supplementary Table 2). At the *OsALS*-TS5 site, both AncBE4max and hyAncBE4max exhibited high editing efficiency at positions C6 (87.5 vs. 77.8%, respectively), C7 (87.5 vs. 55.6%), and C8 (37.5 vs. 55.6%) (Figure 1F and Supplementary Table 2).

The aforementioned results suggest that the editing window for hyAncBE4max was C5-C16, compared to C2-C10 for AncBE4max. Within the C4-C8 editing window, the average C-to-T efficiency for hyAncBE4max was similar or lower than AncBE4max (Figure 1G). The hyAncBE4max performed better at positions C10-C16, especially close to the PAM (Figure 1G). The homozygous rate, percentage of edited plants with homozygous mutations, was similar between AncBE4max and hyAncBE4max at the five target sites studied (Supplementary Table 3). Both systems performed better for TC motifs and poorly for GC motifs, in accordance with previous reports, due to the context preference of the APOBEC1 deaminase (Figures 1B–F; Komor et al., 2016; Gehrke et al., 2018; Thuronyi et al., 2019).

Unexpectedly, the incidence of indels was high for both AncBE4max and hyAncBE4max (24.7 and 35.0% respectively) (Supplementary Figure 1A). Both systems also generated C-to-G substitution byproducts at a relatively high rate (6.14% for AncBE4max and 7.72% for hyAncBE4max) (Supplementary Figure 2A).

### The Addition of the Human Rad51 DNA-Binding Domain Expands the Editing Window of AncBE4max-NG

**FIGURE 2:**
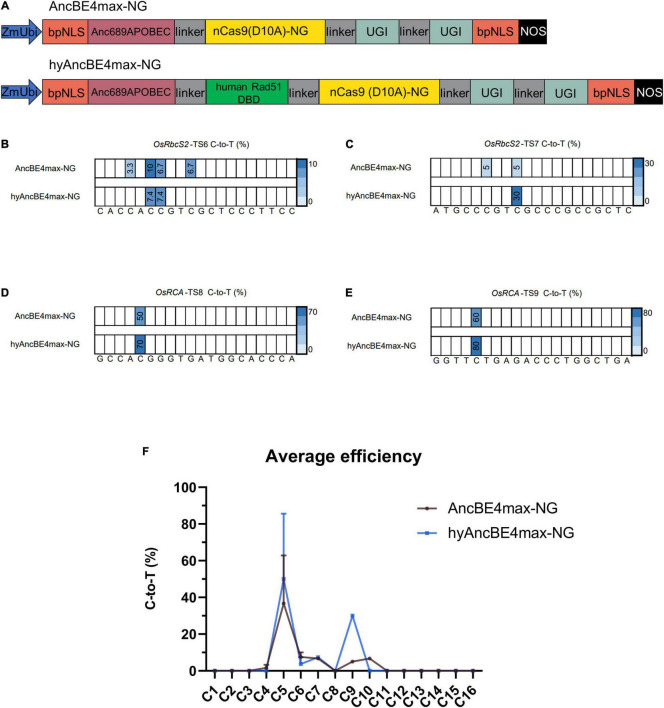
Characterization of AncBE4max-NG and hyAncBE4max-NG in rice. **(A)** Schematic structures of AncBE4max-NG and hyAncBE4max-NG. The human Rad51 DBD was synthesized using a plant optimized code and cloned into AncBE4max-NG (Wang et al., 2019) to form hyAncBE4max-NG. ZmUbi, maize *ubiquitin-1* promoter; bpNLS, bipartite nuclear localization signal; nCas9(D10A)-NG, nickase Cas9-NG with D10A substitution; UGI, uracil glycosylase inhibitor; NOS, NOS terminator. **(B–E)** C-to-T conversions of four target sites using AncBE4max-NG or hyAncBE4max-NG. **(F)** Average C-to-T editing efficiency for AncBE4max-NG and hyAncBE4max-NG based on all four target sites. Data are means ± SD.

SpCas9-NG can recognize NG PAMs expanding the number of target sites compared to the canonical NGG PAM but their C-to-T editing efficiency is much lower than SpCas9-NGG (Nishimasu et al., 2018; Zeng et al., 2020a). We attempted to increase the editing efficiency of SpCas9-NG by fusing the human Rad51 DBD to AncBE4max-NG (Figure 2A). Four target sites from three genes were used to test the editing characteristics of the hyAncBE4max-NG system (Figures 2B–E and Supplementary Table 2).

At the *OsRbcS2*-TS6 site, AncBE4max-NG and hyAncBE4max-NG exhibited similar low editing efficiency at positions C6 (10.0 vs. 7.4%) and C7 (6.7 vs. 7.4%) (Figure 2B). AncBE4max-NG editing efficiency was also low at C4 (3.3%) and C10 (6.7%) while hyAncBE4max-NG displayed no edit activity at either position (Figure 2B). At the *OsRbcS2*-TS7 site, hyAncBE4max-NG exhibited higher efficiency than AncBE4max-NG at position C9 (30 vs. 5%) (Figure 2C); however, only AncBE4max-NG displayed activity at position C6 (5%) (Figure 2C). At the *OsRCA*-TS8 and *OsRCA*-TS9 sites, hyAncBE4max-NG exhibited higher efficiency than AncBE4max-NG at position C5 (70 vs. 50% for *OsRCA*-TS8 and 80 vs. 60% for *OsRCA*-TS9) (Figures 2D,E). As for the AncBE4max system, the average C-to-T efficiencies of hyAncBE4max-NG and AncBE4max-NG in the center of the editing window (C4-C8) were similar (Figure 2F). But, contrary to our expectations, the hyAncBE4max-NG editing window (C5-C9) was narrower than BE4max-NG (C4-C10) (Figure 2F).

The average incidence of indels for hyAncBE4max-NG and AncBE4max-NG was much lower than that observed in the AncBE4max and hyAncBE4max system, with 2.3% for AncBE4max-NG and 0.93% for hyAncBE4max-NG (Supplementary Figure 1B). Interestingly, both hyAncBE4max-NG and AncBE4max-NG exhibited a 30% C-to-G rate at the *OsRCA*-TS9 site, the highest C-to-G rate among all nine target sites (Supplementary Figure 2B).

### Testing the Effect of the Rice Rad51 DNA-Binding Domain on the AncBE4max and evoFERNY Cytidine Deaminases

**FIGURE 3:**
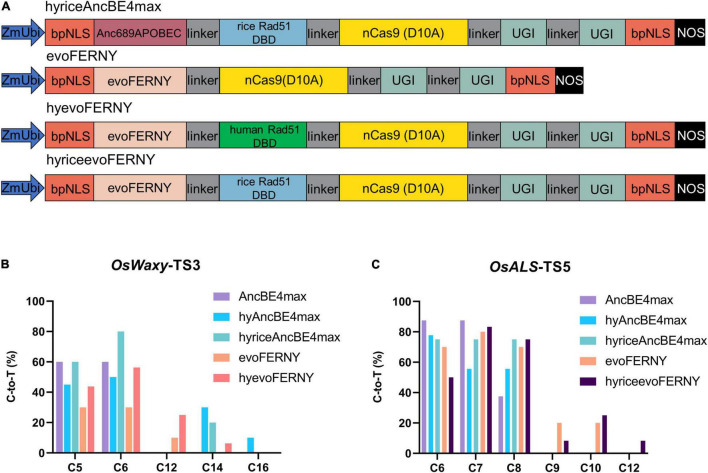
Characterization of hyriceAncBE4max, evoFERNY, hyevoFERNY, and hyriceevoFERNY in rice. **(A)** Schematic structures of hyriceAncBE4max, evoFERNY, hyevoFERNY, and hyriceevoFERNY. Rice Rad51 DBD was synthesized and cloned into AncBE4max to form hyriceAncBE4max. The cytosine deaminase evoFERNY was synthesized using a plant optimized code and cloned replacing Anc689APOBEC in AncBE4max to generate evoFERNY. HyevoFERNY and hyriceevoFERNY were produced by introducing the human Rad51 DBD or rice Rad51 DBD with evoFERNY respectively. **(B)** C-to-T editing efficiency at different positions for *OsWaxy*-TS3 using different base editors. **(C)** C-to-T editing efficiency at different positions for *OsALS*-TS5 using different base editors.

Since the human Rad51 DBD did not have the desired effect on the hyAncBE4max and hyAncBE4max-NG systems, we replaced it with the rice Rad51 DBD (Supplementary Figure 3), to generate hyriceAncBE4max (Figure 3A). Recently, the cytidine deaminase evoFERNY was produced using the phage-assisted continuous evolution system and exhibited superior performance in editing efficiency, target scope, and target context compatibility in mammalian cells and rice (Thuronyi et al., 2019; Zeng et al., 2020b). We combined evoFERNY with either human Rad51 DBD or rice Rad51 DBD to generate hyevoFERNY and hyriceevoFERNY, respectively (Figure 3A).

The editing characteristics for the aforementioned hyCBE base editors were tested at *OsWaxy*-TS3 and *OsALS*-TS5 sites. The editing efficiency of hyriceAncBE4max (60%) was equal to that of AncBE4max (60%) and better than hyAncBE4max (45%) at position C5 of *OsWaxy*-TS3 (Figure 3B and Supplementary Table 2). At position C6, hyriceAncBE4max exhibited higher efficiency (80%) than both AncBE4max (60%) and hyAncBE4max (50%) (Figure 3B). Similar to hyAncBE4max, hyriceAncBE4max also expanded the editing window to C14 at this target (Figure 3B and Supplementary Table 2). At the *OsALS*-TS5 site, hyriceAncBE4max (75%) displayed similar high editing efficiency to hyAncBE4max (77.8%) and AncBE4max (87.5%) at position C6 and outperformed hyAncBE4max (75 vs. 55.6%) at position C7 (Figure 3C and Supplementary Table 2). At position C8, hyriceAncBE4max exhibited higher C to T editing efficiency (75%) than hyAncBE4max (55.6%) and AncBE4max (37.5%) (Figure 3C). Overall, hyriceAncBE4max showed similar or better editing efficiency than hyAncBE4max at these two target sites and it also expanded the editing window.

Compared with AncBE4max, the evoFERNY system produced lower editing efficiencies at positions C5 and C6 of *OsWaxy*-TS3 and positions C6 and C7 of *OsALS*-TS5 (Figures 3B,C). HyevoFERNY displayed higher editing efficiencies than evoFERNY at positions C5, C6, and C12 (43.8, 56.3, 25% for hyevoFERNY and 30, 30, 10% for evoFERNY) and expanded the editing window from C12 to C14 at the *OsWaxy*-TS3 site (Figure 3B). Compared with evoFERNY, hyriceevoFERNY, containing the rice Rad51 DBD, exhibited lower editing efficiencies at positions C6 (50 vs. 70%) and C9 (8.3 vs. 20%) of the *OsALS*-TS5 target site, and similar editing efficiencies at positions C7 (83.3 vs. 80%), C8 (75 vs. 70%), and C10 (25 vs. 20%). The editing window of hyriceevoFERNY also expanded to C12 compared with C10 of evoFERNY (Figure 3C).

Results of this study show that fusion of a human Rad51 DBD or rice Rad51 DBD to either AncBE4max or evoFERNY can broaden the editing window, but the effect on editing efficiency was not satisfactory. Overall, the rice Rad51 DBD outperformed its human counterpart in rice.

## Discussion

Compared to the development of new deaminase variants, which mostly improves base editing efficiency, a fusion of base editors with ssDNA binding protein domains (DBD) has the potential to expand the editing window and improve the editing efficiency given that the DBD can stabilize the Cas9-nickase-generated ssDNA. This strategy has been successfully used in both mammalian cell lines and mouse embryos (Zhang et al., 2020). In addition, a fusion of the Rad51 DBD allowed editing of target sites that could not be edited using conventional base editors in rice (Xu et al., 2022). Since the Rad51 DBD is a non-sequence-specific protein, it has also been used to stabilize the ssDNA and improve the efficiency in the Primer Editor (PE) system (Song et al., 2021).

In this study, the effect of adding DBDs to CBE systems in rice, by fusing the plant optimized-code human Rad51 DBD to the CBE base editors AncBE4max, AncBE4max-NG, and evoFERNY, and the rice Rad51 DBD to AncBE4max and evoFERNY was studied. The results show that the addition of Rad51 DBD had different effects when fused to different Cas9 variants. The human Rad51 DBD improved editing efficiency over the original system in 3 out of 4 target sites when fused to the Cas9-NG AncBE4max-NG, while fusion to the Cas9-NGG AncBE4max resulted in similar or lower editing efficiencies in 4 out of 5 target sites (Figures 1, 2). The moderate or lack of increase in editing efficiency observed in our experiments is in open contrast with the results from Zhang et al. (2020), which reported up to 257-fold increases in editing activity in mammalian systems. Xu et al. (2022) recently tested the effect of the Rad51 DBD in the editing efficiency of a CBE system (Nm1-enBE3) containing the *Neisseria meningitidis* Cas9 and the rat APOBEC1 deaminase in rice. Nm1-enBE3 failed to produce base editing in any of the six targets used in the study while the addition of the Rad51 DBD resulted in low editing efficiencies in 5 of the six targets and failed to produce any editing in the sixth target. Combined, these results and our results suggest that the Rad51 DBD effect on editing efficiency is more accentuated in mammalian systems compared to plant systems, although it could improve plant CBEs with very low efficiency.

Recently, the addition of the human Rad51 DBD to ABE systems produced diverse results in editing efficiency when fused to different Cas9 variants (Tan et al., 2022).

Consistent with previous reports, it was observed that fusion of the Rad51 DBD expanded the editing window to nucleotides close to PAM. The editing window was expanded from position C10 to C16 in hyAncBE4max and from the position, C12 to C14 in hyevoFERNY (Figures 1G, 3C). This study results suggest that hyCBEs performed better at sequences proximal to the PAM. At position C9 of *OsRbcS2*-TS7, the hyAncBE4max-NG exhibited an editing efficiency of 30% compared to 5% for AncBE4max-NG (Figure 2C). Similarly, at position C6 of *OsWaxy*-TS2, hyAncBE4max and AncBE4max exhibited comparable editing efficiencies (55 vs. 50%) but hyAncBE4max outperformed AncBE4max at position C10 (25 vs. 10%) (Figure 3C). At the *OsALS*-TS5 target, the editing efficiency for hyAncBE4max was lower than AncBE4max at positions C6 (77.8 vs. 87.5%) and C7 (55.6 vs. 87.5%). However, hyAncBE4max (55.6%) performed better than AncBE4max (37.5%) at position C8 (Figure 1F). Interestingly, Rad51 DBD narrowed the editing windows for PAM-distal nucleotides. For AncBE4max, at the *OsWaxy*-TS2 and *OsWaxy*-TS4 target sites, the editing window started at C2 and C4, respectively, while AncBE4max-NG displayed activity at position C4 for the *OsRbcS2*-TS6 and *OsRbcS2*-TS7 (Figures 1C,E, 2B,C). However, the corresponding hyCBEs failed to edit at any of the sites. These results suggest that the Rad51 DBD may have adverse effects on the binding or the activity of the cytidine deaminase for PAM-distal sequences.

It has been reported that the DBD had different effects when fused to the rat APOBEC1 and the human APOBEC3A cytidine deaminases in HEK293T cells (Zhang et al., 2020). Fusion of the human Rad51 DBD to the cytidine deaminases Anc689APOBEC and evoFERNY in this study had a similar effect at the *OsWaxy*-TS3 and *OsALS*-TS5 sites (Figures 3B,C). The rice Rad51 DBD performed better than its human homolog when fused to AncBE4max and evoFERNY.

Since the human Rad51 did not increase the off-target effects in mammalian cell lines and mouse embryos, to evaluate the effect of Rad51 on off-targeting in rice, we used CRISPR-GE to search for the homologous sequences within two nucleotides mismatch relative to the sgRNAs and found four putative off-target sites (Xie et al., 2017; Zhang et al., 2020). The editing events, including C to T substitution, C to G substitution, and indels, were undetected at one putative off-target site which contained two nucleotides mismatch within the seed region relative to *OsRbcS2*-TS1 (Supplementary Table 4). For the other three putative off-target sites, generally, the hyCBEs system exhibited similar or even lower off-target efficiency than the CBE system, which indicated that Rad51 also did not increase the off-target effects in rice (Supplementary Figure 4 and Supplementary Table 4).

For the *OsRbcS2*-TS1, *OsWaxy*-TS2 and *OsWaxy*-TS3 targets, both AncCBEmax and hyAncCBEmax showed higher indels (40.1, 40, and 30% for AncCBEmax; 50, 35, and 40% for hyAncCBEmax) (Supplementary Figure 1A). For the *OsWaxy*-TS4 target, hyAncCBEmax contained the indels with 50%, while no indels occurred for AncCBEmax at this target. Conversely, at target *OsALS*-TS5, the indels were 12.5% for hyAncCBEmax, while no indels occurred for hyAncCBEmax (Supplementary Figure 1A). For the NG system, the indels only occurred at *OsRbcS2*-TS6 (3.7%) for hyAncCBEmax-NG and at *OsRCA*-TS9 (30%) for AncCBEmax (Supplementary Figure 1B). In the evoFERNY and hyevoFERNY systems, no indels occurred. Our results suggest that the incidence of indels is related to the nature of the cytidine deaminase, but further research is needed to draw this conclusion.

In summary, we have tested the effects of Rad51 DBD in CBE systems in rice and proved that it can extend the editing window for PAM-proximal sequences, providing advantages for gene functional research and precision molecular breeding, especially for gene saturation mutagenesis.

## Data Availability Statement

The original contributions presented in the study are included in the article/Supplementary Material, further inquiries can be directed to the corresponding author.

## Author Contributions

HZ and CWe designed the work. HZ and CWa supervised the work and analyzed the data. CWe, HL, and PL generated all constructs. CWe, HL, WW, QC, and RL performed the genotyping analysis. HZ and CWe analyzed the data. HZ, CWe, CWa, and JB wrote the manuscript. All authors contributed to the article and approved the submitted version.

## Conflict of Interest

The authors declare that the research was conducted in the absence of any commercial or financial relationships that could be construed as a potential conflict of interest.

## Publisher’s Note

All claims expressed in this article are solely those of the authors and do not necessarily represent those of their affiliated organizations, or those of the publisher, the editors and the reviewers. Any product that may be evaluated in this article, or claim that may be made by its manufacturer, is not guaranteed or endorsed by the publisher.

## Footnotes

1
https://www.genscript.com.cn/gensmart-free-gene-codon-optimization.html
